# Pharmacogenetics-Guided Phase I Study of Capecitabine on an Intermittent Schedule in Patients with Advanced or Metastatic Solid Tumours

**DOI:** 10.1038/srep27826

**Published:** 2016-06-14

**Authors:** Ross Andrew Soo, Nicholas Syn, Soo-Chin Lee, Lingzhi Wang, Xn-Yii Lim, Marie Loh, Sing-Huang Tan, Ying-Kiat Zee, Andrea Li-Ann Wong, Benjamin Chuah, Daniel Chan, Siew-Eng Lim, Boon-Cher Goh, Richie Soong, Wei-Peng Yong

**Affiliations:** 1 Department of Haematology-Oncology National University, Cancer Institute 1E Kent Ridge Road, NUHS Tower Block, Level 7, 119228 Singapore; 2 Cancer Science Institute of Singapore National University of Singapore Centre for Translational Medicine, 14 Medical Drive, #12-01, 117599 Singapore; 3 Translational Laboratory in Genetic Medicine Agency for Science, Technology and Research (A*STAR), Singapore 8A Biomedical Grove Immunos Level 5, 138648 Singapore; 4 Department of Pharmacology Yong Loo Lin School of Medicine National University of Singapore, 21 Lower Kent Ridge Road, 119077 Singapore; 5 Department of Pathology National University Health System National University of Singapore, Lower Kent Ridge Road, 119077 Singapore

## Abstract

The FDA-approved starting dosage of capecitabine is 1,250 mg/m^2^, and market research indicates that U.S. physicians routinely prescribe 1,000 mg/m^2^. Retrospective analyses however report reduced toxicity and efficacy in a subset of patients with the 3R/3R genotype of the thymidylate synthase gene enhancer region (*TSER*). This study sought to develop *TSER* genotype-specific guidelines for capecitabine dosing. Capecitabine was dose-escalated in advanced and/or metastatic cancer patients with *TSER* 3R/3R (Group A; *N* = 18) or 2R/2R + 2R/3R (Group B; *N* = 5) from 1,250 to 1,625 mg/m^2^ b.i.d., every 2 weeks on/1 week off for up to 8 cycles. Parent and metabolites pharmacokinetics, adverse events, and tumour response were assessed. The maximum tolerated and recommended doses in 3R/3R patients are 1,625 mg/m^2^ and 1,500 mg/m^2^. At 1,500 mg/m^2^, one in nine 3R/3R patients experienced a dose-limiting toxicity. Dosing guidelines for *2R/2R* + *2R/3R* remain undetermined due to poor accrual. The results indicate that 3R/3R patients may be amenable to 1,500 mg/m^2^ b.i.d. on an intermittent schedule, and is the first to prospectively validate the utility of *TSER* pharmacogenetic-testing before capecitabine treatment.

In the era of precision oncology, many candidate genetic biomarkers are continuously being identified to be potentially associated with treatment outcomes to commonly used agents in cancer chemotherapy. However, only a handful of pharmacogenetic variants have been prospectively incorporated into dose-finding clinical trials to validate their utility towards therapeutic drug monitoring. Capecitabine is a third-generation orally administered fluoropyrimidine widely used in combination or monotherapy for the treatment of metastatic breast cancer, advanced colorectal and esophagogastric cancer in the adjuvant and metastatic settings whether in first-line or subsequent lines of therapy; and has been shown to downstage patients with resectable rectal cancer in the neoadjuvant setting.

As in the case of many compounds used in chemotherapy, capecitabine demonstrates marked inter-patient and inter-regional variations in toxicity profiles^1^. Haller *et al*. demonstrated from a retrospective pooled analysis of three randomised phase III trials of capecitabine-containing regimens, which comprised 3,053 colorectal cancer patients, that the relative risk for grade 3/4 treatment-related adverse events was lowest in East Asian patients compared to US and European patients^2^. The cause for this inter-regional difference is still a matter of debate, but pharmacogenetics and pharmacoethnicity are likely to play a part.

*TYMS* is a pharmacogene which encodes thymidylate synthase (TS) – the intracellular target enzyme for 5-fluorouracil (5-FU). The promoter enhancer region (TSER) contains a variable number of tandem repeats (VNTR) polymorphism of a 28-base pair sequence, usually occurring in duplet (*2R*) or triplet (*3R*) form, with the *3R* allele being associated with increased TS expression^3^. The *3R/3R* genotype is predominant in Asian populations, and is approximately twice as common in Chinese subjects (67%) compared to Caucasian subjects (38%)^4^.

In earlier studies of fluorouracil-based chemotherapy, grade 3 or 4 toxicity rates of 43–63%, 18–32% and 3–27% were reported for patients carrying the *2R/2R*, *2R/3R* and *3R/3R* genotypes respectively^5^,^6^. A multicentre observational study which prospectively enrolled and genotyped patients treated with fluorouracil monotherapy demonstrated that the proportional odds ratio (OR) for *2R/2R* patients was 1.6 (95% confidence interval, CI: 1.08 to 2.22, *P* = 0.02) on an ordinal scale of global toxicity grade (WHO criteria) in multivariable analysis^7^. In a recent meta-analysis of 1,300 patients, the per-allele odds ratio (OR) for each additional copy of the *2R* allele was 1.36 (95% CI: 1.15 to 1.60, *P* < 0.001) for grade 3 or higher global toxicity^8^. The presence of the *2R* allele however appears to be associated with better clinical response, with studies involving 5-FU-based chemotherapy showing evidence of higher response rates^6^,^9^ and overall survival^9^,^10^.

Based on these evidences, we conducted a genotype-guided phase I study to determine the maximum tolerable dose (MTD) and the recommended phase II dose (RP2D) of capecitabine, and to characterise the safety and feasibility of the regimen. Secondary objectives were to characterise the pharmacokinetics of capecitabine and its metabolites.

## Results

Twenty-three patients with *TSER 3R/3R (n* = 18), *2R/3R (n* = 3) and *2R/2R (n* = 2) were identified and enrolled. In Group B, enrolment was closed early due to slow accrual of patients with the required genotypes. The median turnaround time for TYMS genotyping was 1 day (interquartile range, 1.75 days). The mean ± standard deviation of creatinine clearance was 104 ± 31 mL/min/1.73 m^2^. Additional patient characteristics are given in Table 1. A colorectal cancer patient from Group B treated at dose level 1 (DL1) developed bone metastasis during cycle 1 and was replaced. The median number of treatment cycles was 4.

### Pharmacokinetics of Capecitabine and Its Metabolites

**Figure 1:**
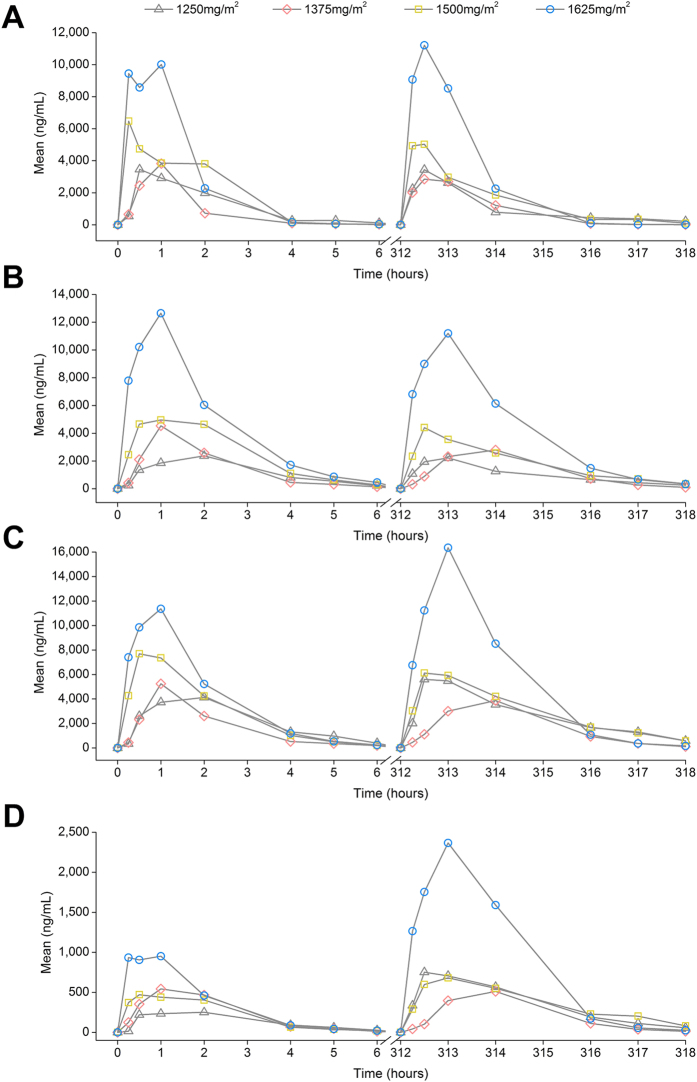
Mean concentration-time profiles of capecitabine and its metabolites. (**A**) Capecitabine; (**B**) 5′-DFCR; (**C**) 5′-DFUR and; (**D**) 5-FU.

**Figure 2:**
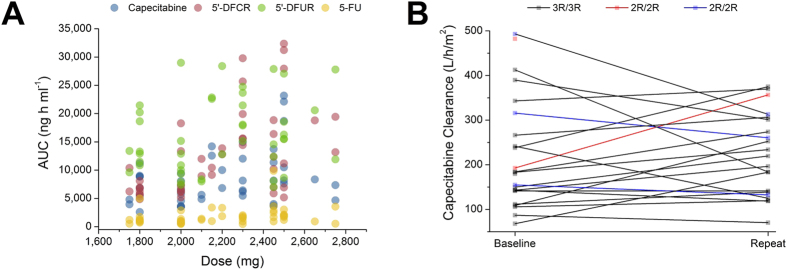
Analysis of capecitabine and its metabolites pharmacokinetics. (**A**) Dose-AUC relationship of capecitabine, 5′-DFCR, 5′-DFUR and 5-FU; (**B**) Variability of capecitabine clearance between first and second pharmacokinetic measurements.

Summary pharmacokinetics of capecitabine and its metabolites are listed in Table 2. Twenty-one patients were evaluated for pharmacokinetics (PK) on both occasions, while two patients did not complete the repeat PK assessment, yielding 44 PK profiles for analysis. None of the patients received modified doses immediately prior to repeat PK ie. all patients received the full amount of the assigned dosage for the repeat PK measurement. Plasma concentration-time profiles of capecitabine and its metabolites are shown in Fig. 1. High interpatient and intrapatient variability in the pharmacokinetics of capecitabine and its metabolites was observed. The inclusion of an interoccasion variability (IOV) parameter for the elimination rate constant (K_el_) for capecitabine, 5′-deoxy-5-fluorocytidine (5′-DFCR) and 5′-deoxy-5-fluorouridine (5′-DFUR) did not significantly improve model fitting, therefore only the fixed effects were calculated. Over the range of doses delivered, capecitabine and its metabolites pharmacokinetics were linear except for the area under the concentration-time curve (AUC) of 5-fluorouracil (5-FU; *P* = 0.1002, linear regression; Fig. 2).

### Toxicity & Feasibility

**Figure 3:**
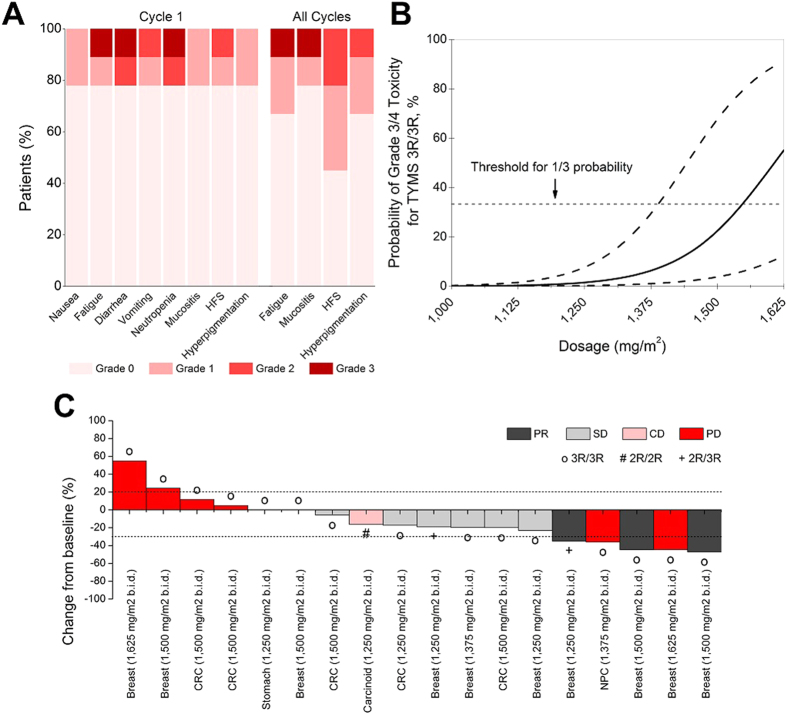
Pharmacogenetic analysis of toxicity and response. (**A**) Characterisation of selected treatment-related adverse events in TYMS 3R/3R patients treated at 1,500 mg/m^2^ during the first cycle or all cycles. (**B**) Per-cycle probability of a 65-year old individual on intermittent schedule capecitabine with *TYMS 3R/3R* genotype experiencing global grade ≥3 toxicity. Dashed curved lines represent 95% confidence interval. Dotted horizontal line represents cut-off probability, wherein maximum tolerable dose is declared if more than one of three patients experienced dose-limiting toxicities. (**C**) Relative change (%) in sum of target lesion size from baseline at best overall response in eighteen evaluable patients. Target lesions were not measured in 2 patients, and tumour assessment at the end of cycle 2 was not completed for 3 patients as a result of clinical deterioration or development of new lesions. Dotted lines indicate RECIST cut-off for partial response (−30%) and progressive disease (+20%). The TYMS genotype for patients are also annotated: 3R/3R (o), 2R/2R (#), 2R/3R (+). Abbreviations: HFS, hand-foot syndrome; PR, partial response; SD, stable disease; CD, clinical deterioration; PD, progressive disease; b.i.d., twice daily.

In Group B, no patients entered at DL1 experienced dose-limiting toxicities (DLTs) during cycle 1. One patient from Group B did not complete toxicity evaluation for cycle 1 because of rapidly progressive metastatic disease and was not included in this analysis. In group A, hand-foot syndrome (HFS) was a frequent late-onset toxicity which occurred in seven patients throughout the study period, of whom only one patient experienced HFS (grade 1) during cycle 1 (Table 3). None of the patients experienced severe (grade ≥3) HFS. No grade 4 toxicities were observed. Severe adverse events (SAEs) were primarily limited to twice daily dosing of 1500 and 1625 mg/m^2^ in Group A, with four of eighteen patients experiencing 9 SAEs. The only grade 3 treatment-related adverse event reported in Group B was enterocolitis, which occurred in a 74 year old Chinese male who was entered at DL1. In cycle 1, DLTs experienced in Group A were neutropenia (DL3 and DL4), mucositis (DL4), and diarrhea (DL4), all of grade 3 severity. DL4 was declared the MTD, and a total of nine patients carrying the 3R/3R genotype were treated at DL3, which is the recommended phase II dose. A total of two (of nine) patients with the *TSER 3R/3R* genotype treated at DL3 experienced grade 3 toxicities during cycle 1: One patient among the first 6 treated patients simultaneously experienced grade 3 neutropenia (DLT) and diarrhea, while another patient from the additional expansion cohort experienced grade 3 fatigue (Fig. 3A).

To estimate the per-cycle probability of grade ≥3 global toxicity, a generalised linear mixed model was used to integrate rich, longitudinal data from repeated measurements of graded toxicity beyond just the first cycle (Fig. 3B). In this model, every 125 mg/m^2^ increase in capecitabine dosage was associated with a 4.2-fold (95% confidence interval, CI: 1.4 to 13.0, *P* = 0.012) increase in odds of grade 3 or higher toxicity after adjusting for age (Odds ratio, OR: 1.004362, 95% CI: 0.934121 to 1.079884, *P* = 0.91) and treatment group (Group A OR: 0.567, 95% CI: 0.020 to 11.9, *P* = 0.66). Correspondingly, the per-cycle probability of any grade ≥3 toxicity in *3R/3R* patients treated at dose-levels 3 and 4 are estimated to be 23% and 55% respectively, corroborating the recommended phase II dose (RP2D) of 1,500 mg/m^2^.

### Tumour Response

Tumour response was evaluated in eighteen patients (Fig. 3C). Target lesions were not measured in two patients, and three patients were not evaluated because of early withdrawal due to clinical deterioration (n = 2) or development of new lesions (n = 1; bone metastasis in a patient with *2R/2R* genotype). No complete responses were observed, but 5 (27.8%) patients had >30% shrinkage in tumour burden of target lesions at best response (Fig. 3). However, this was synchronously accompanied by progressive disease (PD) outside the target lesions in two patients. Nine (50%) patients exhibited stable disease (SD) of ≥6 weeks duration, of which, one patient with carcinoid tumour subsequently discontinued treatment due to worsening pleural effusion without progression of target lesions. The overall median percentage change in the sum of diameters of tumour lesions at best response from baseline was −18.1% (range −47.1–55.0%), and −17.2% (range 47.1–55.0%), −5.9% (range −19.8–11.7%) and −17.2% (range −47.1–55.0%) when only breast cancer, colorectal cancer and *TSER 3R/3R* patients were considered. All six (33.3%) patients with disease progression had the 3R/3R genotype. Aside from 1 patient whose target lesion was not measured, the remaining three patients with *2R/3R* and *2R/3R* genotype exhibited either SD or PR as measured by the Response Evaluation Criteria In Solid Tumours (RECIST).

## Discussion

The US Food & Drug Administration (FDA)-approved starting dosage for capecitabine monotherapy is 1,250 mg/m^2^ b.i.d on a 2 weeks on/1 week off regimen. The results of this genotype-stratified phase I dose-finding study however suggests that a higher dosage is feasible in a subgroup of patients (Group A ie. patients with *TSER 3R/3R*) who exhibit enhanced tolerance to capecitabine and may potentially benefit from higher dose intensities: one in nine patients experienced a DLT at 1,500 mg/m^2^. In any event, the higher MTD achieved in *3R/3R* patients underscores the potential role of genetic biomarkers in tailoring anticancer strategies to the individual’s genetic make-up.

The dose of capecitabine used globally varies according to region. Whilst the approved dosage is widely used in Europe, market research by Hoffmann-La Roche found that physicians in the United States routinely prescribe a lower dosage of 1,000 mg/m^2^ 2, probably because it is thought to have a more favourable therapeutic index^11^. Disparities in the given dosage may also partly reflect differences in the goals of chemotherapy, cross-cultural differences in perceived tolerability, or pharmacogenetics/pharmacoethnicity. Cross-ethnic comparative studies have also shown that regional differences in dose requirements, tolerability or efficacy of various oncological drugs exists^12^,^13^,^14^. The MTD derived from western phase I studies are not necessarily transmutable to Asian patient cohorts because of differences in the underlying phenotypic distributions of the populations^12^. For example, our group had previously contrasted the efficacy and pharmacokinetics-pharmacodynamics of docetaxel and S-1, another fluoropyrimidine, between Asians and Caucasians^15^,^16^. It is also worth highlighting that the starting dosage of docetaxel is 60 mg/m^2^ every 3 weeks in Japan, compared to 75–100 mg/m^2^ in western countries^17^.

To our knowledge, irinotecan is the only other chemotherapeutic agent which has revisited phase I dose-escalation trials for the purpose of devising genotype-guided dosage recommendations^18^. In this study, it was hypothesized that patients without the *UGT1A1* *28/*28 genotype would tolerate higher doses of irinotecan. Fifty-nine white patients with either the *UGT1A1* *1/*1 or the *1/*28 genotype underwent dose-escalation of irinotecan. *UGT1A1* *28/*28 patients were not eligible. With the exclusion of patients with the *UGT1A1* *28/*28 genotype, MTD was declared at 370 mg/m^2^ and 310 mg/m^2^ in *1/*1 and *1/*28 patients, which is considerably higher than the recommended dose of 180 mg/m^2^ in the FOLFIRI regimen. In the present study, the recommended dose for *TSER 3R/3R* patients was found to be 1,500 mg/m^2^ when dose escalation was conducted separately from *2R/2R* and *2R/3R* patients. These evidence suggests that greater adoption of phase I study designs which stratify patients by genotype or ethnicity could work towards the favour of precision medicine.

In this study, the grouping of *TSER 2R/2R* and *2R/3R* into a single arm in this study was determined *a priori*, as it was conducted in an Asian setting where the prevalence of these genotypes are far less common as compared to populations of Caucasian ancestry, and accrual was expected to be modest at best^4^. For this practical limitation, it was not an objective of this study to risk-stratify patients with *TSER 2R/2R* and *2R/3R* genotypes. Another limitation was not a study objective to evaluate differences in response rates between treatment groups due to disease heterogeneity and limited patient numbers, which precluded meaningful comparisons from being performed. Therefore, the clinical efficacy of a higher dosage will have to be evaluated in a follow-up phase II study preferably involving a more homogenous patient cohort.

Interestingly, it was recently suggested that the associations of the TSER polymorphism with fluoropyrimidine toxicity could in fact be due to variants in ENOSF1, an adjacent gene which is poorly characterised. Rosmarin and colleagues reported that rs2612091 or rs2741171 of ENOSF1, which are in linkage disequilibrium with *TSER 2R/3R* and the TYMS 3′-untranslated region 6 bp ins-del polymorphism, statistically associates better with capecitabine-related HFS than the TYMS polymorphisms^19^. Therefore, if a functional mechanism underlying the rs2612091/rs2741171 associations is found, then the TYMS 5′VNTR polymorphism may actually be an incidental, surrogate biomarker. We anticipate that ongoing molecular biological studies would elucidate the role of ENOSF1 in FU-based therapy towards the goal of safer and more effective chemotherapy dosing.

To summarise, we conducted a genotype-guided dose escalation trial and established a recommended phase II dose of 1,500 mg/m^2^ for patients with the *TSER 3R/3R* genotype. This prospective study significantly adds to the growing evidence base for pharmacogenetic testing of the TSER genotype prior to capecitabine and FU-based regimens.

## Patients and Methods

This was an open-labelled dose escalation study at the National University Hospital, Singapore. The study protocol was approved by the Domain Specific Review Board, National Healthcare Group, Singapore, and the study was carried out in accordance with the approved guidelines. All patients provided written, informed consent prior to study entry. The study was registered in ClinicalTrials.gov on June 11, 2008 under the registration number NCT00697502.

### Eligibility

Patients with a histologically or cytologically proven advanced or metastatic non-haematological malignancy were eligible for this study provided that they had failed previous lines of systemic treatment, or had tumours for which no standard treatment options exist or capecitabine was indicated. Other eligibility criteria were as follows: *TSER 2R/2R*, *2R/3R* or *3R/3R* genotypes; age ≥21 and <70 years; performance status of at least 70% (Karnorfsky) or <2 (Eastern Cooperative Oncology Group); evaluable and/or measurable disease according to the Response Evaluation Criteria In Solid Tumours (RECIST); life expectancy of at least 3 months; absolute neutrophil count ≥1500/μL; platelets ≥100,000/μL; total bilirubin ≤1.5 times the upper limit of normal; aspartate transaminase (AST), alanine transaminase (ALT) and alkaline phosphatase ≤2.5 times the upper limit of normal (or 5 times, if hepatic metastases were present). Patients were not eligible if they had: received any anticancer therapy within 28 days of drug administration or had not recovered from side effects of prior treatment; creatinine clearance less than 50 mL/min/1.73 m^2^; known dihydropyrimidine deficiency; malabsorption or conditions that would impair gastrointestinal uptake; prior treatment with capecitabine. Other antineoplastic agents, investigational drugs or immunotherapy was not allowed during the study.

### Treatment and Dose Escalation

Capecitabine was given orally twice daily for 14 days followed by a 7-day rest period for 8 cycles. Patients were segregated depending on TSER genotype into Group A (*3R/3R*) or Group B (*2R/2R* and *2R/3R*). The starting dose in both groups was 1250 mg/m^2^ twice daily, which was increased to 1375, 1500, 1625 and 1750 mg/m^2^. A pharmacist was responsible for communicating the dose to the treating physicians, who were blinded to the patient genotype status and subgroup.

Dose limiting toxicity (DLT) was defined as any drug-related grade 3 or greater toxicity, with the exception of reversible transaminitis, nausea, vomiting, alopecia and grade 3 skin toxicity which resolves to grade ≤2 within 7 days and does not cause capecitabine to be withheld for more than four doses during the first cycle. Three patients were enrolled at each dose level, and in the absence of DLTs, the dose was escalated, and three additional patients were treated at the next dose level. If one patient developed DLT, three further patients will be treated at that level, and dose escalation continued if no additional DLTs occurred. MTD was declared at the dose preceding that for which more than one of the three or six patients had DLTs, and another three recruited patients would be treated at the MTD to better characterise its pharmacokinetic and safety profile. The last patient at a dose level must have completed the first cycle and been evaluated before the first patient of the next dose level can be entered. Intrapatient dose escalation was not allowed.

### Dose Modifications and Supportive Measures

If any grade 2 toxicity occurred, capecitabine was withheld until resolution to grade ≤1 and restarted at the same dose level. Treatment was withheld for recurrence of grade 2 toxicity, or for grade 3 non-hematologic or grade 4 hematologic toxicity until resolution to grade 1 or less, and resumed at the preceding dose level for the subsequent cycle. Treatment was discontinued if any grade 4 non-hematologic toxicities occurred. Nausea, vomiting and diarrhoea were promptly treated with best supportive care. Granulocyte colony-stimulating factor was only given to treat febrile neutropenia.

### Clinical Assessment

A complete medical history was collected during screening. Physical examination, evaluation of performance status, full blood counts and chemistries was done at baseline and before each course of treatment. Adverse events were graded according to the National Cancer Institute-Common Toxicity Criteria (v3.0) at the end of every cycle. Computed tomography scans of tumour lesions was performed at baseline and repeated every two cycles, and tumour response was graded using the RECIST criteria. Study compliance was recorded in the patient case record file.

### Pharmacokinetics

Serial blood samples (5 mL) were drawn into heparin-containing vacutainers on days 1 and 14 of cycle 1 at the following time points: predose, and 0.25, 0.5, 1, 2, 4, 5 and 6 hours after administration. Blood samples were centrifuged at 1,500 *g* for 10 minutes at 4 °C, supernatant plasma collected, and stored at −20 °C until analysis. The concentration of capecitabine and its metabolites (5′-DFCR, 5′-DFUR and 5-FU) was assayed using a validated multiplex liquid chromatography-mass spectrometry/mass spectrometry (LC-MS/MS) method in negative mode as previously described^20^. The LC-MS/MS system consisted of an API 4000 triple-quadrupole mass spectrometer (Applied Biosystems/MDS SCIEX, Ontario, Canada) and an Agilent 1100 series binary pump, degasser and autosampler (Agilent Technologies, Waldbronn, Germany). Capecitabine and its metabolites were separated in reverse-phase on an Alltima C_18_ column (150 mm × 2.1 mm, inside diameter 5 μm; Alltech Applied Science, Breda, the Netherlands). The mobile phase solvents were Milli-Q water and high-performance liquid chromatography (HPLC)-grade acetonitrile, delivered at a constant rate of 350 μL/min over a run time of 12 min. The linear gradient setting was used, and the mobile phase composition changed from 1% to 20% over the first 3 minutes, and to 95% over the next 5 minutes, before rapid reversion to 1% over 6 seconds.

The following PK parameters for capecitabine and its metabolites were estimated using noncompartmental methods (WinNonlin; Pharsight, Munich Germany): area under the plasma concentration time curve from 0 to infinity (AUC_0-∞_), AUC from time 0 to the last sampling time (AUC_0-t_), maximum plasma concentration (C_max_), time to maximum plasma concentration (T_max)_, half-life (t_1/2_) and elimination rate constant (K_el_). Body surface area (BSA)-normalised apparent total clearance (CL/F) and volume of distribution (Vd/F) were estimated for capecitabine. All BSA-normalised exposure parameters were tested for dose linearity before a dose-normalised, pooled parameter estimate was derived (see Statistics).

### TYMS Genotyping

Germline DNA was isolated from circulating lymphocytes and extracted using the DNeasy Blood and Tissue Kit (Qiagen, Hilden, Germany). Genotyping of the TYMS enhancer region (TSER) variable number of tandem repeats was performed as described previously^21^.

### Statistics

Descriptive statistics were used to summarise PK parameters. Interindividual variability (IIV) and interoccasion variability (IOV) in PK parameters for the overall sample pool were estimated using multilevel model for repeat measures, in which the fixed effects and residual variance represent the inter- and intra-individual variance respectively. IIV and IOV were expressed as coefficients of variation (CV%) by taking the square roots of the estimates divided by the mean parameter value. The per-cycle probability of grade ≥3 global toxicity at a given dose level was estimated using generalised linear mixed model (GLMM) with maximum likelihood estimator^22^,^23^,^24^. GLMM was used as a more robust longitudinal assessment of the RP2D and could factor in subsequent, repeated measurements of graded toxicity apart from the first cycle. For the mixed model, a more stringent per-patient approach which avoids intraclass correlation issues was used, by averaging PK parameters for patients with two PK measurements into a single value^25^. An extended waterfall plot was employed to visualize best overall tumour response^26^. All *P* < 0.05 were deemed nominally statistically significant, while *P* < 0.1 were indicative of trends.

## Additional Information

**How to cite this article**: Soo, R. A. *et al*. Pharmacogenetics-Guided Phase I Study of Capecitabine on an Intermittent Schedule in Patients with Advanced or Metastatic Solid Tumours. *Sci. Rep.* **6**, 27826; doi: 10.1038/srep27826 (2016).

## Figures and Tables

**Table 1:** Patient characteristics.

Age	
Median (range)	58
Range	34–74
Gender	
Female	13 (56.5%)
Male	10 (43.5%)
Ethnicity	
Chinese	17 (73.9%)
Malay	4 (17.4%)
Indian	2 (8.7%)
Primary tumour	
Breast	11 (47.8%)
Colorectal	6 (26.1%)
Laryngeal & nasopharyngeal	2 (8.7%)
Hepatocellular & pancreatic	2 (8.7%)
Gastric & carcinoid	2 (8.7%)
Disease sites (target + non-target)	
Median	5
Range	1–12
Prior treatment	
Chemotherapy	
No previous treatment	3 (13.0%)
1 regimen	10 (43.5%)
2 regimens	3 (13.0%)
≥3 regimens	7 (30.4%)
Hormonal therapy	9 (39.1%)
Surgery	15 (65.2%)
Radiotherapy	13 (56.5%)
Creatinine clearance (mL/min/1.73 m^2^)	
Mean ± SD	106 ± 30.9

**Table 2:** Pharmacokinetics.

Day	Capecitabine Dose (mg/m^2^)								Overall*
	1250		1375		1500		1625		
	1	14	1	14	1	14	1	14	
No. patients (*3R3R*/*2R3R* + *2R2R*)	7 (3/4)	6 (3/3)	4 (3/1)	4 (3/1)	9 (9/0)	8 (8/0)	3 (3/0)	3 (3/0)	44 (35/9)
Capecitabine									
Cmax (ng ml^−1^)	6147 (73%)	7543 (96%)	3858 (40%)	3388 (61%)	12301 (76%)	7226 (80%)	13017 (21%)	12587 (71%)	3.662 (44%/71%)
Tmax (h)^†^	1 (0.25–2)	0.75 (0.25–4)	1 (0.5–1)	1 (0.2–2)	1 (0.25–2)	1 (0.25–4)	0.75 (0.25–1)	1 (0.5–1)	(0.25–4)
T_1/2_ (h)	1.13 (39%)	1.43 (51%)	1.01 (30%)	0.766 (22%)	0.722 (33%)	1.05 (75%)	0.87 (42%)	0.85 (22%)	21%/51%)
AUC_τ_ (ng h ml^−1^)	6594 (37%)	6140 (36%)	4438 (17%)	4817 (31%)	9843 (53%)	7938 (34%)	14962 (20%)	14436 (48%)	3.657 (37%/36%)
AUC_∞_ (ng h ml^−1^)	6858 (39%)	6388 (37%)	4493 (19%)	4825 (31%)	9869 (53%)	8224 (32%)	15009 (20%)	14450 (47%)	3.758 (37%/35%)
CL/F (L h^−1 ^m^−2^)	226 (53%)	211 (39%)	366 (25%)	340 (9.70%)	194 (50%)	201 (30%)	113 (20%)	147 (53%)	7%/32%)
Vd/F (L m^−2^)	360 (63%)	376 (29%)	508 (21%)	370 (17.6%)	196 (49%)	303 (75%)	153 (55%)	188 (67%)	3%/43%)
Kel (h^−1^)	0.69 (31%)	0.60 (40%)	0.74 (28%)	0.949 (21%)	1.05 (27%)	0.88 (38%)	1.04 (57%)	0.86 (26%)	0.85 (40%)a
5′-DFCR									
Cmax (ng ml^−1^)	4276 (52%)	5040 (92%)	4683 (37%)	5665 (79%)	9035 (59%)	6077 (90%)	13067 (31%)	12063 (38%)	3.072 (47%/50%)
Tmax (h)^†^	2 (0.5–4)	0.75 (0.25–4)	1 (1–2)	1.5 (1–2)	1 (0.25–2)	1 (0.5–4)	1 (0.5–1)	1 (1–2)	1 (0.25–4)
T_1/2_ (h)	0.98 (21%)	1.22 (60%)	1.21 (31%)	0.85 (16%)	1.06 (38%)	1.26 (79%)	0.97 (24%)	0.87 (14%)	1.08 (54%)a
AUC_∞_ (ng h ml^−1^)	7949 (33%)	8223 (56%)	8691 (30%)	10367 (53%)	14599 (45%)	11203 (28%)	26994 (22%)	24433 (25%)	5.601 (45%/26%)
Kel (h^−1^)	0.74 (21%)	0.71 (34%)	0.67 (44%)	0.84 (18%)	0.75 (34%)	0.76 (40%)	0.77 (28%)	0.81 (14%)	0.75 (32%)a
5′-DFUR									
Cmax (ng ml^−1^)	6644 (46%)	11645 (67%)	5433 (35%)	8783 (86%)	11712 (71%)	9163 (62%)	12907 (41%)	17707 (54%)	4.537 (41%/55%)
Tmax (h)^†^	2 (1–4)	1.5 (0.5–4)	1 (1–2)	1.5 (1–2)	1 (0.25–4)	1.5 (0.5–4)	1 (0.5–2)	1 (1–2)	1 (0.25–4)
T_1/2_ (h)^†^	1.09 (48%)	1.02 (41%)	1.30 (45%)	0.71 (16%)	0.92 (39%)	0.82 (35%)	0.80 (18%)	0.77 (14%)	0.94 (19%/40%)
AUC_∞_ (ng h ml^−1^)	12921 (23%)	18076 (34%)	9682 (18%)	14237 (56%)	16754 (38%)	17509 (38%)	22708 (23%)	29857 (55%)	7.683 (29%/36%)
Kel (h^−1^)	0.74 (30%)	0.78 (34%)	0.65 (45%)	1.01 (15%)	0.84 (29%)	0.95 (33%)	0.90 (21%)	0.92 (15%)	0.84 (32%)a
5-FU									
Cmax (ng ml^−1^)	454 (60%)	1424 (73%)	623 (81%)	1024 (74%)	908 (76%)	1008 (45%)	1254 (61%)	2541 (60%)	0.4657 (36%/77%)
Tmax (h)^†^	2 (0.25–4)	0.75 (0.25–4)	1 (1–2)	1.5 (1–2)	1 (0.25–2)	1.5 (0.5–4)	1 (0.25–2)	1 (1–2)	1 (0.25–4)
T_1/2_ (h)	1.22 (51%)	0.96 (35%)	1.12 (34%)	0.85 (42%)	1.31 (42%)	1.24 (31%)	1.07 (8%)	0.90 (25%)	1.13 (23%/34%)
AUC_∞_ (ng h ml^−1^)	816 (26%)	2238 (61%)	1303 (103%)	1808 (58%)	1242 (41%)	2205 (33%)	2021 (34%)	4915 (72%)	b
Kel (h^−1^)	0.70 (39%)	0.81 (30%)	0.73 (45%)	0.99 (42%)	0.63 (40%)	0.62 (35%)	0.65 (8%)	0.84 (31%)	0.72 (26%/30%)

*Data is presented as mean (IIV%/IOV%). Exposure parameters, Cmax and AUC, are dose (mg^−1^) and BSA-normalised (m^−2^).

^†^Median (range).

^a^Coefficient of variation was not separated into IIV and IOV components as likelihood ratio test indicated no significant (*P* < 0.05) improvement in model fitting over a linear regression model with fixed effects only.

^b^Combined exposure parameter value not calculated due to lack of significant (*P* < 0.05, linear regression) dose-proportional relationship.

Abbreviations: Cmax, maximum concentration; Tmax, time to maximum concentration; T_1/2_, half-life; AUC_τ_, area under concentration-time curve from time 0 to last sampling time; AUC∞, area under the concentration-time curve from 0 to infinity; CL/F, apparent total clearance; Vd/F, apparent volume of distribution; Kel, elimination rate constant; 5′-DFCR, 5′-deoxy-5-fluorocytidine; 5′-DFUR, 5′-deoxy-5-fluorouridine; 5-FU, 5-fluorouracil; IIV, interindividual variability; IOV, interoccasion variability; BSA, body surface area.

**Table 3:** Adverse Events.

	No. of patients (%)					
	*3R/3R* (n = 18)			*2R/2R* + *2R/3R* (n = 5†)		
	Grade 1	Grade 2	Grade 3	Grade 1	Grade 2	Grade 3
Cycle 1 only						
Nausea	2	0	0	1	0	0
Fatigue	2	0	1	0	0	0
Diarrhea	1	1	2	1	0	0
Vomiting	3	2	0	0	1	0
Anorexia	1	0	0	0	0	0
Myalgia	0	0	0	1	0	0
Gastritis	0	1		0	0	0
Mucositis	2	0	1	0	0	0
Neutropenia	0	2	2	0	0	0
Hand-foot syndrome	1	1	0	0	0	0
Rash	1	0	0	0	0	0
Dry Skin	1	0	0	0	0	0
Hyperpigmentation	3	0	0	0	0	0
All Cycles						
Nausea	4	0	0	1	0	0
Fatigue	4	0	2	0	0	0
Diarrhea	1	1	2	1	0	0
Vomiting	4	2	0	0	1	0
Anorexia	2	0	0	0	0	0
Dyspepsia	1	0	0	0	0	0
Enterocolitis	0	0	0	0	0	1
Abdominal Pain	1	0	0	0	0	0
Myalgia	0	0	0	1	0	0
Gastritis	0	1	0	0	0	0
Mucositis	1	0	2	0	0	0
Neutropenia	0	2	2	0	1	0
Hand-foot syndrome	4	3	0	0	0	0
Rash	1	0	0	0	0	0
Dry Skin	2	0	0	0	0	0
Hyperpigmentation	3	2	0	2	0	0
Dental abscess	0	0	1	0	0	0
Peripheral neuropathy	1	0	0	0	0	0
Dehydration	0	1	0	0	0	0

^†^One patient did not complete toxicity evaluation for cycle 1 due to rapidly progressive metastatic disease.
